# Association of Differentiation-Related Gene-1 (DRG1) with Breast Cancer Survival and *in Vitro* Impact of DRG1 Suppression

**DOI:** 10.3390/cancers4030658

**Published:** 2012-07-10

**Authors:** Ruqia Mehmood Baig, Andrew J. Sanders, Mahmood Akhtar Kayani, Wen G. Jiang

**Affiliations:** 1 Metastasis and Angiogenesis Research Group, Institute of Cancer and Genetics, Cardiff University School of Medicine, Cardiff CF14 4XN, UK; 2 Cancer Genetics Lab, COMSATS Institute of Information Technology, Islamabad 44000, Pakistan

**Keywords:** DRG1, breast cancer, metastasis, prognosis

## Abstract

Differentiation-related gene-1, DRG1, is a metastasis suppressor gene whose expression has been shown to be dysregulated in a number of malignancies. The current study examines the expression of DRG1 in a clinical breast cohort and its association with a number of clinical pathological factors using quantitative polymerase chain reaction. Additionally, DRG1 expression is targeted *in vitro* using ribozyme transgene technology to explore the function of DRG1 in two human breast cancer cell lines. Low levels of DRG1 were found in patients who developed metastasis (*p* = 0.036) and who died of breast cancer (*p* = 0.0048) compared to disease free patients. Knockdown of DRG1 also resulted in significantly increased invasion and motility, but decreased matrix-adhesion in MCF7 cells. Knockdown of DRG1 seemed to have minimal impact on the cellular functions of the MDA-MB-231 breast cancer cell line causing no significant differences in cell growth, invasion, motility or matrix-adhesion. Thus, DRG1 appears to be linked to development of metastasis and death in patients who died as a result of breast cancer and may be useful as a prognostic factor as its knockdown appears to be linked with increased invasion and motility and decreased adhesion in MCF7 breast cancer cells.

## 1. Introduction

Metastasis suppressor genes are defined by their capacity to control metastatic dissemination without affecting growth of the primary tumour. The complexity of the metastatic process suggests that it is controlled at the genetic level via the activation and/or deactivation of multiple genes. The first of these metastasis suppressor genes was identified in 1988 [1]. These genes play their role by inhibiting the spread of primary tumour cells, either by arresting them at the primary site, or by affecting their invasion, adhesion or extravasations in the surrounding environment [2].

Differentiation-related gene-1, DRG1, is a metastasis suppressor gene originally identified by differential display as being significantly up regulated by induction of differentiation in colon carcinoma cells *in vitro* [3]. DRG1 mRNA encodes a 43 kDa protein and has been found to be expressed in all tissues examined, with the highest expression seen in the brain, prostate, kidney, and intestine [3,4]. The expression of DRG1 appears to be regulated by multiple signals, including hypoxia, androgen, homocysteine, Ni^2+^, *N*-Myc and PTEN [5,6,7,8,9,10,11,12]. DRG1 has been shown to be upregulated by iron chelators in a variety of cancer cell lines [13]. In addition, the tumour suppressor p53 and von Hippel-Lindau factor have been shown to modulate DRG1 gene expression *in vitro* [14,15]. DRG1 expression has also been shown to be down-regulated in malignancy. An inverse correlation has been reported between DRG1 expression and the metastatic potential of prostate cancer cells. Low DRG1 expression correlates with higher grade of prostate cancer, development of metastases, and with poor patient survival [7,16]. The metastasis suppressor activity of DRG1 has also been observed in colon cancer cells *in vivo *and *in vitro* [4]. Kurdistani *et al.* showed that introduction of DRG1 cDNA in human bladder cancer cells suppresses tumuorigenicity in nude mice [14]. In colorectal cancer, it has been suggested that DRG1 expression may be associated with a less aggressive, indolent colorectal cancer. However, high expression of DRG1 also appears to be associated with relative resistance to irinotecan chemotherapy [17]. In breast cancer, low DRG1 expression is associated with more advanced cancer stage and worse survival [18]. Inhibition of DRG1 expression resulted in signiﬁcant changes in microtubule structure and the disappearance of α-tubulin protein. Therefore, it has been suggested that the loss of DRG1 may contribute to genomic instability in cancer cells [19]. A large number of studies have identiﬁed DRG1 as a gene that is down regulated in cancer and associated with metastasis suppression, however there are some studies that suggest differing roles. Some studies have indicated that DRG1 expression is up regulated in prostate cancer compared with corresponding normal tissue but that this could be due to its response to hormones such as androgens [20]. These studies imply that the loss of hormone-dependence is responsible for the previously observed decrease of DRG1 expression in some cancers [21]. Interestingly, a study examining DRG1 expression in African-American prostate cancer patients has found that they have significantly reduced expression of this protein when compared with Caucasian prostate cancer patients [22]. While there is some controversy regarding the relationship of DRG1 expression to the suppression of metastasis in certain cancers, the majority of studies have demonstrated that higher levels lead to a less aggressive phenotype [21]. Many studies have implied that a loss of DRG1 expression results in a more aggressive, metastatic phenotype and identify DRG1 as a potential prognostic indicator.

The aim of the current study was to investigate the expression profile of DRG1 in a cohort of breast cancer patients and compare this expression profile with clinical outcomes of patients in the cohort. Additionally, this study aimed to explore the role of DRG1 on cellular functions such as growth, adhesion, invasion and cellular migration in breast cancer cell lines, using a series of *in vitro* cell models.

## 2. Experimental Section

### 2.1. Cell Lines and Culture Conditions

MDA-MB-231 and MCF7 cell lines were purchased from the American Type Culture Collection (ATCC, Rockville, MD, USA) and maintained in Dulbecco’s modified Eagles medium (DMEM) supplemented with 10% foetal calf serum (PAA Laboratories Ltd., Somerset, UK), streptomycin and penicillin. Cells were incubated at 37 °C, 5% CO_2_ and 95% humidity.

### 2.2. Patient Samples and Preparation

Breast cancer tissues (n = 114) and normal background tissues (n = 31) were collected immediately after surgery and stored in the deep freezer at −80 °C. Patients were routinely followed clinically after surgery. The median follow-up period was 120 months. The presence of tumour cells in the collected tissues was verified by examination of frozen sections following H & E staining by a consultant pathologist. Clinical details of the patients are outlined in Table 1.

**Table 1:** Clinical and pathological information of the breast cancer cohort.

Clinical data		Sample numbers
***Grade***		
	1	20
	2	38
	3	54
***TNM***		
	1	60
	2	40
	3	7
	4	4
***NPI***		
	1	57
	2	38
	3	15
***Outcome***		
	Disease free	80
	With metastasis	7
	With local recurrence	5
	Died of breast cancer	14

### 2.3. DRG1 Knockdown in Human Breast Cancer Cell Lines

DRG1 was found to be expressed in both MCF7 and MDA-MB-231 breast cancer cell lines at the transcriptional level using conventional primers outlined in Table 2. Hammerhead ribozyme transgenes, specifically targeted to DRG1 transcripts were constructed based on the secondary structure of DRG1 mRNA. Following design and synthesis by Invitrogen (Paisley, UK), the ribozymes were first amplified using the respective oligos (Table 2) using touch down PCR and were cloned into a mammalian pEF6/His TOPO vector (Invitrogen) and transfected into MCF7 and MDA-MB-231 cells, as previously reported [23,24]. MCF7 and MDA-MB-231 cells were also transfected with an empty pEF6 control plasmid. Cells were then subjected to selection with blasticidin (5 µg/mL) and then subsequently maintained in media containing 0.5 µg/mL blasticidin. This process allowed the generation of stably transfected MCF7 and MDA-MB-231 cells containing the ribozyme transgene and expressing reduced DRG1 levels.

**Table 2:** Primer sequences used in the study.

Primer	Forward Primers	Reverse primers
DRG1 Conventional	GGACGATTTCACAAAAACAT	CATCTTCATACTGCAAAGCA
DRG1 Quantitative	TGCTACAGCTGATGACCTC	ACTGAACCTGACCTGACCGTA CACCAATTCCTCAATGGAGAT
GAPDH Conventional	GGCTGCTTTTAACTCTGGTA	GACTGTGGTCATGAGTCCTT
GAPDH Quantitative	CTGAGTACGTCGTGGAGTC	ACTGAACCTGACCGTACACAGAGA TGATGACCCTTTTG
DRG1 Ribozyme1	CTGCAGCAGTGTTGACTTCCCCACACTGATGAGTCCGTGAGGA	ACTAGTGATGCTCGAATTGGATTTGTTGGTTTTCCATTTCGTCCTCACGGACT
DRG1 Ribozyme2	CTGCAGGACATCCAGAACAATCAACTGATGAGTCCGTGAGGA	ACTAGTGGCCCGAACCTGTAACTTGATTTCGTCCTCACGGACT

MCF7 and MDA-MB-231 cells containing the control pEF6 plasmid were designated as MCF7^pEF6^ and MDA-MB-231^pEF6^ and those containing the ribozyme transgenes were labelled MCF7^DRG1KD^ and MDA-MB-231^DRG1KD^. Wild type cells without any plasmid and transgene were called MCF7^WT^ and MDA-MB-231^WT^. Suppression of DRG1 expression in MCF7^DRG1KD^ and MDA-MB-231^DRG1KD^ cells was verified, in comparison to MCF7^pEF6^ and MDA-MB-231^pEF6^ and wild type, using reverse transcription-polymerase chain reaction (RT-PCR) and Western blot analysis.

### 2.4. RNA Extraction and Reverse Transcription-Polymerase Chain Reaction

RNA was extracted using total RNA isolation reagent in accordance with the provided protocol (TRI reagent, Sigma, Dorset, UK). Sample RNA was quantified using a spectrophotometer (WPA UV 1101, Biotech Photometer, Cambridge, UK) and standardized to a concentration of 500 ng. This RNA was used as a template to reverse transcribe cDNA using an iScript cDNA synthesis kit (Bio Rad Laboratories, Hemel Hempstead, UK). Following cDNA synthesis, samples were probed using glyceraldehyde 3-phosphate dehydrogenase (GAPDH) primers to check cDNA quality and confirm uniform sample cDNA levels, together with those specific for DRG1 transcript (full primer details are shown in Table 2). Polymerase chain reaction (PCR) was performed in a T-Cy Thermocycler (Creacon Technologies Ltd., Emmen, The Netherlands) using REDTaq^®^ ReadyMix™ PCR Reaction Mix (Sigma). The PCR reaction consisted of an initial denaturing step of 94 °C for 5 min, 30 cycles of 94 °C for 40 s, 55 °C for 40 s and 72 °C for 1 min and a final extension step of 72 °C for 10 min before holding at 4 °C. PCR products were separated on an agarose gel, stained with ethidium bromide and visualized under ultraviolet light.

### 2.5. Quantitative-Polymerase Chain Reaction (Q-PCR)

Real-time quantitative PCR was also used to assess DRG1 transcript levels as previously reported [25]. Results are given as number of transcripts/μL based on an internal standard and the results were further normalised using the expression of GAPDH in these samples. The Q-PCR technique used the Amplifluor system (Intergen Inc., New York, NY, USA), Q-PCR Master Mix (ABgene, Surrey, UK) and a universal probe (Uniprimer™, Intergen) to record the fluorescence emitted during the reaction. Conditions for Q-PCR were: an initial 15 min 95 °C period followed by 60 cycles of 95 °C for 15 s, 55 °C for 60 s and 72 °C for 20 s. Full details of primers used are given in Table 2.

### 2.6. SDS-PAGE and Western Blotting

Cell pellets were lysed with an SDS lysis buffer for 1 h, followed by removal of insolubles after centrifugation at 13,000 rpm. Cell lysates were later quantified using the Bio-Rad DC Protein Assay kit (Bio-Rad Laboratories). Denatured samples with equal protein concentrations were separated using SDS PAGE gels. Proteins were blotted onto nitrocellulose membrane, following which they were probed for specific proteins following the SNAP ID protocol provided (Millipore, Watford, UK). GAPDH was used as internal control. The protein bands were subsequently visualized using the Supersignal TM West Dura system (Pierce Biotechnology, Rockford, IL, USA). DRG1 antibody, raised in goat (Santa Cruz Biotechnologies, Santa Cruz, CA, USA) and GAPDH, raised in mouse (Santa Cruz Biotechnologies), were used for probing at a concentration of 1:100 and 1:200 respectively. Anti-goat and anti-mouse secondary antibodies were obtained from Sigma and used at a 1:350 concentration in line with recommended settings outlined in the SNAP ID protocol.

### 2.7. *In Vitro* Growth Assay

The effect of DRG1 suppression on MCF7 and MDA-MB-231 cell growth rates was assessed using an *in vitro* growth assay. Cells were seeded at a density of 3,000 cells per well, in 200 μL of medium, into 96 well plates. Triplicate plates were set up and incubated for overnight, 3-day and 5-day periods before analysis. Following incubation, the plates were fixed in 4% formaldehyde (v/v) and stained with 0.5% (w/v) crystal violet and then treated with 10% acetic acid (v/v), prior to colorimetric detection of cell density by spectrophotometric analysis at 540 nm using a Bio-Tek ELx800 multi-plate reader (Bio-Tek Instruments Inc., Winooski, VT, USA).

### 2.8. *In Vitro* Matrigel Adhesion Assay

This was based on a previously published protocol [25]. In brief, Matrigel™ basement membrane matrix purchased from BD Biosciences (Oxford, UK), was used to pre coat 96-well plates with a 5 µg per well layer. 45,000 cells were added per well in 200 μL of medium. After incubation for 45 min, wells were vigorously washed with BSS to remove unbound cells. Adherent cells were then fixed in 4% formaldehyde (v/v) and stained with 0.5% (w/v) crystal violet. Stained cells were later counted in a number of random fields under a ×20 objective.

### 2.9. Electric Cell-Substrate Impedance Sensing Analysis of Breast Cancer Cell Lines Migration

The electric cell-substrate impedance sensing (ECIS) system (Applied Biophysics Inc, Troy, NJ, USA) was used to detect and track MCF7 and MDA-MB-231 cell migration as described previously [26,27]. Briefly, 200,000 cells per well in 300 μL of medium containing Hepes buffer were seeded onto ECIS 96W1E arrays and incubated until a confluent monolayer formed over the array electrodes. This monolayer was then wounded electrically to create a simultaneous physical break in the cell monolayer of equal dimensions. Rate of change in impedance, as cells migrated back onto the electrode, was then monitored and measured using the ECIS software provided.

### 2.10. *In Vitro* Matrigel Invasion Assay

Invasion assays were undertaken using inserts (ThinCert™ containing 8 μm pores, Greiner bio-one, Germany) in 24-well tissue culture plates. Each insert was first coated with 50 μg/insert of Matrigel. 20,000 cells were seeded into each insert. After 72 h, cells that had invaded and migrated through the matrix and adhered to the underside of the insert were fixed and stained with 4% (v/v) formaldehyde and 0.5% (w/v) crystal violet following a method previously reported [28].

### 2.11. Statistical Analysis

Experimental procedures were repeated independently at least three times. In all assays the transfected breast cancer cell lines, containing the DRG1 ribozyme transgenes, were compared with respective pEF6 plasmid controls (cells containing closed pEF6 plasmid only) using a two-sample, two-tailed *t*-test. The data values given represent the mean value ± SEM, and values of *p* ≤ 0.05 were considered to be statistically significant.

## 3. Results

### 3.1. Correlation of DRG1 Transcript Expression with Grade, TNM and NPI Status

**Figure 1:**
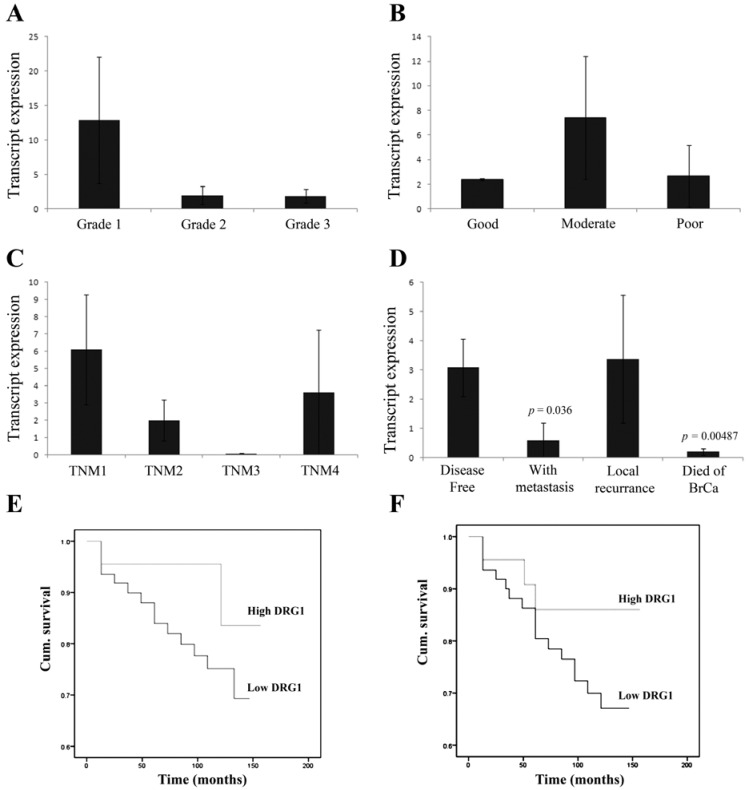
Levels of expression of DRG1 transcripts in breast cancer tissues. No significant differences were seen in DRG1 levels between Grade (**A**); predicted prognosis *i.e*., based on NPI value of each patient (divided into good, moderate and poor) (**B**) and TNM status (**C**); Significant reductions in DRG1 levels were seen in patients who developed metastasis (*p* = 0.036) and those who died of breast cancer (*p* = 0.0048) compared to disease free patients (**D**); However, no significant differences were seen in overall survival (**E**) and disease free survival (**F**) rates of patients who had relatively high or low DRG1 expression levels.

Levels of DRG1 transcript were analyzed in connection with tumour grade. No significant difference in DRG1 transcript levels between grade 1 (well differentiated), grade 2 (moderately differentiated) and grade 3 (poorly differentiated) tissues was observed (Figure 1A). NPI (Nottingham Prognostic Index) was also used as an indicator to assess the relationship between DRG1 transcript and predicted prognosis. Patients were divided into groups *i.e*., good, moderate and poor prognosis according to NPI values. No significant difference was observed among these groups (Figure 1B). Moreover, the relationship between DRG1 expression and TNM status was also analyzed. Similarly, no significant differences in DRG1 transcript expression levels were observed between early stage (TNM1) and later stage (TNM2, 3 or 4) tissues (Figure 1C).

### 3.2. Reduced DRG1 Expression Is Associated with Poorer Patient Prognosis

Patients were divided in relation to clinical outcome at final follow up into patients who remained disease free, those with metastasis, those with local recurrence and those who died of breast cancer. Patients who were disease-free were found to have significantly higher levels of DRG1 transcripts than those who developed metastasis and those who died of breast cancer (*p* = 0.036 and 0.0048 respectively, Figure 1D). Additionally, lower levels of DRG1 tended to be associated with a poorer overall patient survival (Figure 1E) and disease free survival (Figure 1F). Patients with low levels of DRG1 expression had a mean survival time of 122.5 months (95% CI 111.2–133.8) compared to 146.6 months (95% CI 133.5–159.7). Similarly, patients with lower expression levels of DRG1 also had a reduced disease free survival [118.2 months (95% CI 106.4–130.0)] compared to those with higher levels of DRG1 [140.1 months (95% CI 123.2–157.0)]. However, neither overall survival nor disease free survival rates for those with low compared to those patients with high levels of DRG1 was found to be statistically significant (*p* = 0.133 and 0.176 respectively).

### 3.3. Knockdown of DRG1 in Breast Cancer Cell Lines

**Figure 2:**
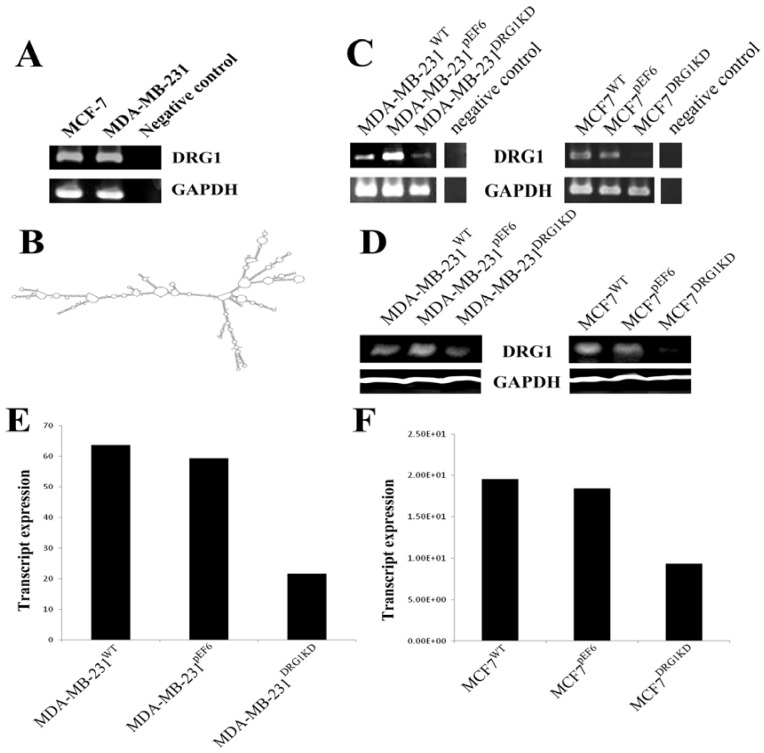
Confirmation of knockdown of DRG1 in MCF7 and MDA-MB-231 human breast cancer cell lines. (**A**) DRG1 expression was observed in both MCF7 and MDA-MB-231 breast cancer cell lines; (**B**) Secondary structure of human DRG1 which was used in the design of anti-DRG1 transgenes; (**C**) Low transcript levels of DRG1 were seen in MCF7^DRG1KD^ and MDA-MB-231^DRG1KD^ cells using RT-PCR; (**D**) Knock down of DRG1 was also observed at the protein level using Western blot analysis. (**E**,**F**) Q-PCR confirmation of DRG1 knockdown in MDA-MB-231 (**E**) and MCF7 (**F**) cells.

DRG1 expression was observed in both the MCF7 and MDA-MB-231 human breast cancer cell lines available (Figure 2A). Ribozyme transgenes, were subsequently designed based on the secondary structure of the DRG1 transcript (Figure 2B), to target DRG1 expression in these cells. Transfection of MCF7 and MDA-MB-231 breast cancer cells with the DRG1 ribozyme transgene successfully knocked down the expression of this molecule (Figure 2C–F). A reduction in DRG1 expression was observed in MCF7^DRG1KD^ and MDA-MB-231^DRG1KD^ compared to the respective wild type (MCF7^WT^ and MDA-MB-231^WT^) and plasmid control (MCF7^pEF6^ and MDA-MB-231^pEF6^) cells (Figure 2C). Quantitative PCR similarly showed successful transcript knock down of DRG1 in MCF7^DRG1KD^ and MDA-MB-231^DRG1KD^ compared to the respective control lines (Figure 2E,F). In keeping with the knock down of transcript expression, reduced protein levels were also observed in MCF7^DRG1KD^ and MDA-MB-231^DRG1KD^, compared to the respective controls, using Western blot analysis (Figure 2D).

### 3.4. Effect of DRG1 Knockdown on the Growth of Breast Cancer Cells

**Figure 3:**
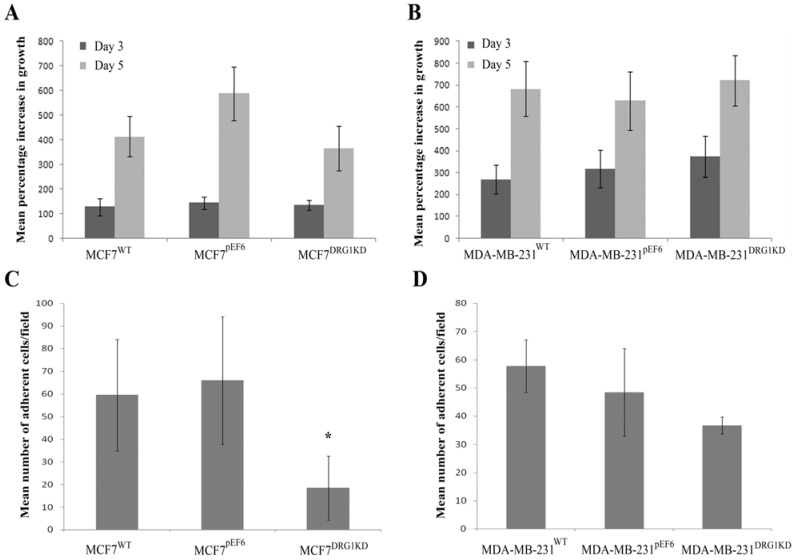
Effect of knockdown of DRG1 on MCF7 and MDA-MB-231 cell growth and matrix-adhesion. DRG1 knockdown did not have any significant effect on cell growth over 3 and 5 day incubation periods (**A**,**B**). However, knockdown of DRG1 could significantly reduce matrix-adhesion in MCF7 cells (*p* = 0.023), though no significant effects were seen in MDA-MB-231 cells (**C**,**D**).

The knockdown of DRG1 expression had little impact on the growth of the tested breast cancer cells over the 3 and 5 day incubation periods. At both time points no significant differences were observed between MCF7^DRG1KD^ or MDA-MB-231^DRG1KD^ and the respective control cells (*p* > 0.05) (Figure 3A,B).

### 3.5. Effect of DRG1 Knockdown on Adhesion of Breast Cancer Cells

Interestingly, knockdown of DRG1 seemed to impact on cell-matrix adhesion differently in the two tested breast cancer cell lines. A significant decrease in adhesiveness was observed between MCF7^DRG1KD^ cells and control MCF7^pEF6^ (*p* = 0.023), while no significant differences were observed between MDA-MB-231^DRG1KD^ and MDA-MB-231^pEF6^ (Figure 3C,D).

### 3.6. Effect of DRG1 Knockdown on Invasion of Breast Cancer Cells

**Figure 4:**
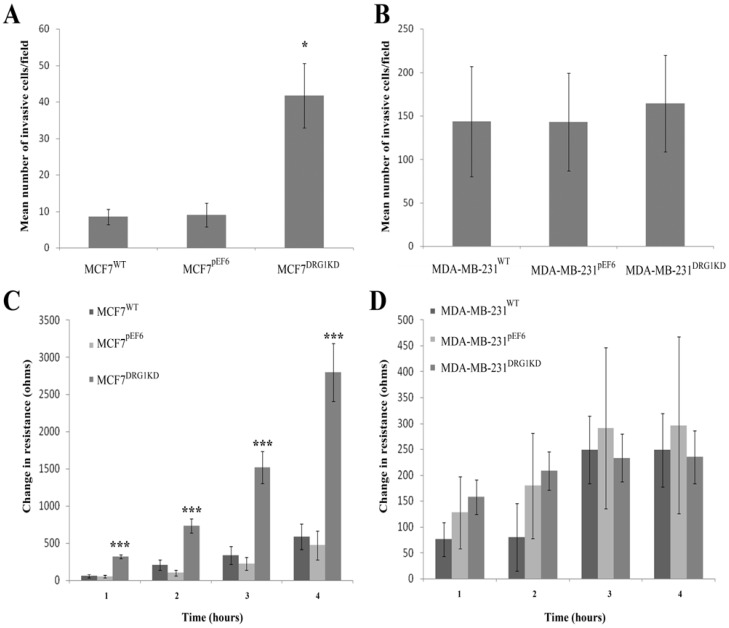
Knockdown of DRG1 significantly enhanced the invasive potential of MCF7 cells (*p* = 0.013), but did not significantly affect MDA-MB-231 cells (**A**,**B**); Similarly, decreased expression of DRG1 significantly increased MCF7 cells migration capacity in an ECIS based migration assay following 1 h (*p* = 0.00007), 2 h (*p* = 0.0002), 3 h (*p* = 0.0004) and 4 h (*p* = 0.0007) periods (**C**); However, knockdown of DRG1 in MDA-MB-231 cells did not significantly impact on their migratory capacity (**D**).

MCF7^DRG1KD^ cells were found to be significantly more invasive, compared to MCF7^pEF6^ cells (*p* = 0.013) in the Matrigel *in vitro* invasion assay. In contrast to this, once again, DRG1 knockdown in MDA-MB-231 cells seemed to have very little impact on cellular invasiveness and MDA-MB-231^DRG1KD^ cells showed no significant change in invasiveness compared with control MDA-MB-231^pEF6^ cells (Figure 4A,B).

### 3.7. Effect of DRG1 Knockdown on Migrational Rates of Breast Cancer Cells

The electric cell substrate impedance sensing (ECIS) system was used to examine the effect of DRG1 knockdown on breast cancer cell migration. Following electrical wounding, MCF7^DRG1KD^ cells, migrated at a much faster rate to re-cover the electrode compare to MCF7^pEF6^ cells as detected by the enhanced rate of change in resistance detected by the ECIS system. In contrast to this, MDA-MB-231^DRG1KD^ cells showed no significant change compared to control cells (Figure 4C,D).

## 4. Discussion

Tumour metastasis is an important clinical problem, contributing to the majority of cancer related deaths. The recent discovery of metastasis suppressor genes, has introduced a novel approach to treating cancer and preventing metastasis. A number of metastasis suppressor genes have been identified in several types of cancer and their association with tumour growth, proliferation and cell motility is an area of investigation [29]. DRG1 has been shown to play an important role in the context of human cancer progression [3,4,14,16,17,18,30,31]. The current study aimed to verify the expression profile of DRG1 in a cohort of breast cancer patients and to compare it with the clinical data. This study also aimed to validate the impact of this gene on several *in vitro *cellular functions and its potential contribution to metastasis.

Our data suggests that DRG1, reported to be a potential suppressor of metastasis, is aberrantly expressed in breast tumour tissues. Levels of DRG1 were found to be correlated with clinical outcomes and long term survival of the patients suffering with breast cancer. Bandyopadhyay, 2004 has previously suggested that the down regulation of DRG1 is at least partially at the RNA level [18]. This is also consistent with a previous report in which DRG1 mRNA was shown to be reduced in a small set of breast and prostate tumours by *in situ* hybridization [14].

Considering the role of DRG1 in metastasis suppression, it was proposed that its expression could be used as a prognostic marker in cancer patients [16,18]. This follows the observation that individuals with higher DRG1 tumour levels have greater survival rates, in breast and prostate cancer patients [16,18]. This is also supported by a study examining DRG1 expression in colorectal cancer and comparing it with patient outcomes [17]. Kaplan-Meier analysis of pancreatic cancer patients showed a statistically significant correlation of DRG1 expression with survival [32]. The clinical data presented in this study suggests, similar to these previous studies, that high DRG1 patient transcript levels may be indicative of a better prognosis, with lower levels of DRG1 expression being associated with metastatic spread and patients who died from the disease. However, we did not observe any significant association in our cohort between DRG1 expression levels and overall or disease free survival. The results of our current study also suggest a potential role of DRG1 in cell adhesion, invasion and motility as knockdown of this molecule in MCF7 breast cancer cells decreased the adhesive ability of this cell line while increasing invasiveness and migratory capacity. However, these effects are not apparent in the MDA-MB-231 breast cancer cell line and additional work will be required to fully understand the mechanism behind these differences, which may lie in the different nature and expression profiles of the two cell lines. One potential explanation for this may be due to the differing expression profiles of the two cell lines, for example it may be that the ER/hormone dependent pathway is involved in this phenomenon as MCF7, which is ER+ and a hormone responsive cell line [33] showed significant results while MDA-MB-231 cells gave no significant results, though this needs to be fully investigated.

The increase in invasiveness in the MCF7 cell line is consistent with findings that suggest that over-expression of DRG1 reduces the invasiveness of breast, colon and prostate cancer cells [4,16,18,32]. The enhanced motility and decreased adhesiveness of DRG1 knockdown MCF7 breast cancer cells demonstrated by this study further suggest a metastasis suppressive role for this gene. Changes in motility, adhesiveness and cellular invasion are key traits, required for tumour progression and the acquisition of metastatic competence [34].

Guan *et al.* found that E-cadherin, an adhesion and metastasis suppressor molecule [35], is up regulated by DRG1 [4]. Increased expression of E-cadherin has also been shown to reduce the motility of metastatic breast cancer cells *in vitro* [35]. However, it is unlikely that E-cadherin is the only molecular target of DRG1 that leads to metastasis suppression. Further studies, assessing the targets and interacting molecules of DRG1, are required to aid in the understanding of the molecular mechanisms of metastasis suppression by this molecule.

## 5. Conclusions

In conclusion, the *in vitro *andclinical data presented here indicate an involvement of the DRG1 gene in breast cancer progression and demonstrate a potential role of this gene in suppressing tumour metastasis.
